# Fractal MapReduce decomposition of sequence alignment

**DOI:** 10.1186/1748-7188-7-12

**Published:** 2012-05-02

**Authors:** Jonas S Almeida, Alexander Grüneberg, Wolfgang Maass, Susana Vinga

**Affiliations:** 1 Div Informatics, Dept Pathology, University of Alabama at Birmingham, USA; 2 Research Center for Intelligent Media, Furtwangen University, Furtwangen, Germany; 3 Information and Service Systems, Dept of Law and Economics, Saarland University, Germany; 4 INESC-ID, Instituto de Engenharia de Sistemas e Computadores, Investigação e Desenvolvimento, Lisboa, Portugal; 5 FCM-UNL Faculdade Ciências Médicas - Universidade Nova de Lisboa, Portugal

## Abstract

**Background:**

The dramatic fall in the cost of genomic sequencing, and the increasing convenience of distributed cloud computing resources, positions the MapReduce coding pattern as a cornerstone of scalable bioinformatics algorithm development. In some cases an algorithm will find a natural distribution via use of *map *functions to process vectorized components, followed by a *reduce *of aggregate intermediate results. However, for some data analysis procedures such as sequence analysis, a more fundamental reformulation may be required.

**Results:**

In this report we describe a solution to sequence comparison that can be thoroughly decomposed into multiple rounds of *map *and *reduce *operations. The route taken makes use of iterated maps, a fractal analysis technique, that has been found to provide a "alignment-free" solution to sequence analysis and comparison. That is, a solution that does not require dynamic programming, relying on a numeric Chaos Game Representation (CGR) data structure. This claim is demonstrated in this report by calculating the length of the longest similar segment by inspecting only the USM coordinates of two analogous units: with no resort to dynamic programming.

**Conclusions:**

The procedure described is an attempt at extreme decomposition and parallelization of sequence alignment in anticipation of a volume of genomic sequence data that cannot be met by current algorithmic frameworks. The solution found is delivered with a browser-based application (webApp), highlighting the browser's emergence as an environment for high performance distributed computing.

**Availability**

Public distribution of accompanying software library with open source and version control at http://usm.github.com. Also available as a webApp through Google Chrome's WebStore http://chrome.google.com/webstore: search with "usm".

## Background

Since 2008 the decrease in sequencing costs is far steeper than of those of computing [1]. Projecting from these trends promises to deliver the $1000 genome by 2014, making it inescapable that the costs of analyzing the raw sequence data will exceed those of its generation. In contrast, the algorithms used to process and compare sequences largely rely on the dynamic programming solutions proposed by Smith-Waterman and Needleman-Wunsch in the 70's and 80's [2,3]. This is not to say that the implementation of alignment algorithms has not become more efficient, quite the opposite has taken place. For example, there are several capable algorithmic solutions [4] to align the vast number of short reads that next generation sequencing techniques produce a reference genome. However, better implementations of dynamic programming do not by themselves remove its limited scalability, which has motivated research into a variety of alignment-free methods in the last decade [5-9].

The efficiency gains in implementation owe some of its advances to a major improvement in parallelization. A particularly valuable development is the support of functional programming patterns that explicitly identify opportunities for parallelization through MapReduce [10]. This development is a major attraction of cloud computing services such as Amazon's Elastic MapReduce (a hosted Hadoop framework) and is turning high performance computing into a commodity [11]. In a nutshell, a map function is one that is applied independently to each element of an array whereas a reduce function is one that aggregates them into a single result. In practice, many implementations of MapReduce use a key emission mechanism to allow for aggregation into multiple results as illustrated in mapreduce-js.googlecode.com. Nevertheless, that higher-level elaboration can be ignored for the purpose of the decomposition described here. In summary, map-reduce functions, now natively supported by many languages, identify opportunities for distribution and parallelization which can be handled automatically by the programming environment without exposure to the procedural overload of message passing interfaces (MPI). For example, considering the case of a numerical array, sum and max are reduce functions whereas internal product is a map function. Accordingly, the use the MapReduce functional pattern now underlie many of the leading genomic analysis packages such as GATK [12] and CloudBurst [13] and is the key cloud computing abstraction for large scale data management and analysis [11,14].

Having parallelization handled at the algorithm identification level creates an opportunity to revisit sequence analysis for additional fragmentation into map-reduce patterns functions. In that regard, conventional alignment using dynamic programming presents a serious obstacle to parallelization because it requires the reprocessing of the symbolic sequences every time a new pair of sequences is considered. Specifically, suffix reuse by dynamic programming locks the analysis of a sequence position to that of the neighboring positions - every time a pair-wise comparison is made. That limitation motivated us to revisit an alignment-free methodology to identify opportunities for a more extreme use of map-reduce patterns in sequence analysis.

The use of iterated maps to represent nucleotide sequences, a fractal projection technique, was introduced by the Chaos Game Representation procedure, CGR, first proposed over two decades ago [15]. The realization that this representation is an order independent Markov transition table was proposed a decade later [16], followed by the Universal Sequence Map (USM) variation on the CGR theme the following year [17], which represents each unit of the sequence with context as a order-free numerical coordinate.

These explorations of iterated maps as order free representation on sequence context led to the labeling of these approaches as being "alignment-free" [5], in the modern sense that they are free from the *reduce *dynamic programming procedure. Numerous applications and advancements have since been proposed with approximately two hundred publications currently referring back to that review. Using the new terminology one could now describe the appeal of alignment-free sequence statistics as described, for example, by [18], as being precisely those of a map function resolved to the individual sequence unit.

## Methods

### CGR and USM

**Figure 1:**
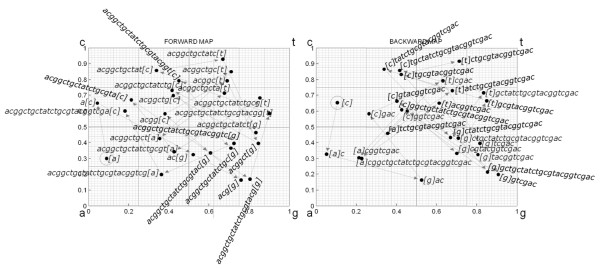
**Graphic computation of USM encoding by generating forward (Equation 1) and backward (Equation 2) CGR successions**. See Table 1 for the numeric representation. The graphic format makes it easy to verify that each position is obtained by moving the coordinates half the distance to the identity edge of the next sequence unit. Note also how the circular seeding, (Figure 2) causes the first coordinate computed for each map to be at half the distance between the last coordinate and the identity edge of the first sequence unit.

The fundamental iteration of the Chaos Game Representation (CGR) technique [15] is that of assigning a numerical coordinate to each symbol of a sequence, calculated as the previous position plus half the distance to the next. This procedure graphically illustrated in the Results section (Figure 1). The Universal Sequence Maps, USM [17], starts with a variation on the CGR theme by expanding it to any vocabulary, and by running the iteration both forward and backward in the sequence (Equation 1 and 2): for a given sequence, S, with *N *units/symbols, *S = s_1_... s_N_, with s_i _∈ A*, A is any alphabet, and with reference to a unit hypercube with h dimensions, with its edges, E, assigned to individual units/symbols of the alphabet, A, in order to assign each symbol, *s_i_*, to a vector-valued coordinate *c_i_= [c_i_^forward^, c_i_^backward^] *by following the procedure described in Equation 1 and 2. This procedure is also demonstrated and illustrated with an example in the Results section.

(1) $$c_{i}^{forward}=c_{i-1}^{forward}+\frac{E\left(i\right)-c_{i-1}^{forward}}{2},\;i=1,\cdots \;,N,\;E\in {\left\{0,1\right\}}^{h}$$

(2) $$c_{i}^{backward}=c_{i+1}^{backward}+\frac{E\left(i\right)-c_{i+1}^{backward}}{2},i=1,\cdots \;,N,\;E\in {\left[\left[0,1\right]\right]}^{h}$$

A number of elaborations on the CGR theme were advanced to produce the USM representation, such as a) seeding the succession as if the sequence was circular instead of starting at the 1/2 coordinate, b) identifying the sequence alphabet to define a unitary hypercube, and c) resolving both forward (Equation 1) and backward (Equation 2) coordinates. For detailed description and discussion of computing scale independent motifs as Universal Sequence Map (USM) coordinates see [19]. For a generalization of the CGR representation without sacrificing the conveniency of a 2D representation see [20].

The critical property of CGR coordinates is that they bijectively map to the symbolic sequence that generated them: each [0, 1]^n ^coordinate corresponds to a unique sequence and each sequence corresponds to a unique [0, 1]^n ^coordinate. The analysis of the CGR/USM projection has been used to derive measures of sequence similarity (dissimilarity distance) directly from the coordinates in many of the reports cited above. While some of these metrics provide a simple algebraic solution to the lower boundary of sequence similarity, here we will use the exact iterated solution [21,22] described in Equation 3, where L (c1, c2) represents the dissimilarity between coordinates c1 and c2, measured as the length of the common prefix:

(3) $$L\left(c1,c2\right)=\left|\begin{array}{c} x=0\\ while\;\left(round\left(c1\cdot 2^{x}\right)==round\left(c2\cdot 2^{x}\right)\right)\left\{x=x+1\right\}\\ return\;x \end{array}\right)$$

The critical improvement of USM over the underlying CGR succession is that one can determine the length of a shared sub-sequence solely by comparing the USM coordinates of any two homologous sequence units. This claim could have been anticipated from the results reported in [17] but its effective realization is only reported here and relies on a map-reduce composition of the procedure described in Equation 3. Careful inspection of the code (usm.js method *L*) will show that the implementation of this formulation is bound by the numerical resolution of the processor to values of L smaller than 64. The practical resolution of this constraint is straightforward and is detailed in the Alignment subsection in Results, under "5. Sequence alignment to full genomes": it requires the recalculation of the value of L at the edges of the 64 similar length segment resolved.

#### MapReduce

The MapReduce algorithm parallelization pattern [10] is inspired on two primitives of functional languages, *map *and *reduce*. The *map *function will process the elements of an array independently, for example [1-4]. *map*(*function*(*x*){*return *2 · *x*}) will produce the result [2,4,6,8]. The reduce function will instead be applied consecutively to consecutive elements of an array. For example, [2,4,6,8]. *reduce*(*function*(*a, b*){*return a + b*}) will add the array elements one by one, by replacing pairs of elements picked in arbitrary sequence by their sum, until only one is left with the value *20*. In contrast to the *map *function which is applied independently to each array element, the *reduce *function is processed iteratively. The MapReduce pattern then articulates the map and reduce functions through the emission of keys: each map function issues one or more keys and each reduce function targets the map results emitted with a specific key, as elegantly illustrated in [23]. In the sequence analysis decomposition described here the emission of keys will be omitted because the procedure is the same in its entirety regardless of the value of the USM coordinates. In other words, the key emitted by the map function would always be the same and therefore there is only one reduce function needed per map operation. The MapReduce pattern is finding increasing use in Bioinformatics [14], with particularly significant applications to sequence analysis [12,13]. There is, therefore, ample infrastructure support for the implementation of the procedure described here.

#### JavaScript

The functional decomposition of sequence analysis described here is best constructed, and verified, in a functional programming environment. This approach has the additional advantage of providing a description of the algorithm that is closer to a mathematical notation [24]. As highlighted in that seminal work, functional descriptions of computational procedures (algorithms) facilitate of their analysis as mathematical objects. An additional criteria in the selection of the programming environment is that it should be readily available to the audience of this report, without requiring the installation of specialized interpreters or other additional software. Finally, it should also be an environment where MapReduce is possible as both a native operation and as a procedure in a distributed computing environment. JavaScript (ECMAScript ISO/IEC 16262) satisfies all of these requirements: as the "assembler language of the web" with an efficient interpreter in every modern browser; it supports code injection natively, removing the need to "install" the libraries provided with this report; it is a functional programming language with native map and reduce Array methods; open source implementations of MapReduce through server side execution of JavaScript are also readily available, for example, as part of open source projects such as Apache Foundation's CouchDB and MongoDB. Accordingly both the accompanying reference libraries and the algorithm descriptions in this report were coded in JavaScript (see Availability).

#### Referencing code and its execution

The algorithm decomposition described in this report is delivered as a JavaScript library and also as a versioned webApp at http://usm.github.com (see Availability). The use of a version control system will also allow referring to specific lines in the code for the version in place at the time of submission of this report (version id 07a39896293a57ecdeec571335ae782bb56c2972). For example, the similar length calculation, *L*, described in Equation 2, at the time corresponded to line 184 of the usm.js, which can be inspecting by following the link https://github.com/usm/usm.github.com/blob/07a39896293a57ecdeec571335ae782bb56c2972/usm.js#L184. For convenience, these links will be treated as literature references "authored" by the corresponding object variable. For example, the link above can be found in the list of references under [25]. The same procedure will allow the reader to load the usm object the way it was at that time by, instead of using the URL https://raw.github.com/usm/usm.github.com/master/usm.js described in the project's home page, specifying the version requested as https://raw.github.com/usm/usm.github.com/07a39896293a57ecdeec571335ae782bb56c2972/u sm.js.

## Results

### Organization of MapReduce decomposition

The MapReduce decomposition of sequence analysis is organized along the chain of procedures performed when two arbitrary sequences are compared. The first step is the encoding of the sequence into a USM "numerical structure" [Vinga 2011]. Second, the encoding procedure is then verified by decoding back to the symbolic sequence. Third, the numerical coordinate based distance calculation is performed. Fourth, all pieces are brought together in a single MapReduce comparison of multiple positions and full sequences.

### Open source library

An open source library, usm.js, is provided with all procedures described here (see Availability). An accompanying interactive webApp that uses that library where the individual components can be tried is also included. Note modern browsers provide access to the command line, details and screencast video demo included in the open source project, so all 4 procedures described above can be engaged directly. For example, *u = new usm*('*acggctgctatctgcgtacggtcgac*') will automatically extract the 'acgt' alphabet and encode the sequence. Individual functions can be used piecewise, for example *u = new usm*()*;u.encode*('*acggctgctatctgcgtacggtcgac*') would have the same effect. The first syntax style will be used here to takes full advantage to JavaScript's functional style by chaining the call to a specific result of the analysis. For example, to extract the alphabet (attribute "abc") one could do

> new usm('acggctgctatctgcgtacggtcgac').abc

"acgt"

### 1. Encoding: Alphabet extraction and map compaction

The first pre-processing step is that of using, or extracting if not specified, the list of unique symbols used in the sequence - the alphabet. That list is then processed to generate the compact coordinates of a hyper-dimensional unitary cube [17]. Illustrating with the example above,

ubase = new usm('acggctgctatctgcgtacggtcgac');ubase.cube

**Figure 2:**
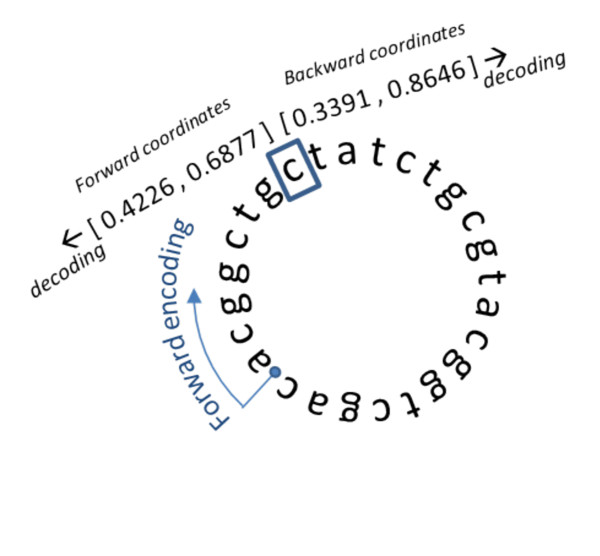
**Encoding and decoding the base sequence**. The period identifies the junction between the beginning and end of the sequence. Forward encoding (Equation 1, Figure 1 Left) takes place clockwise and Backward encoding (Equation 2, Figure 1 right) takes place counterclockwise. Both forward and backward CGR coordinates are displayed for the 8th unit of the sequence. The adjacent sequence units can be determined (decoded) from those coordinate values alone. As shown later, this observation can be used to assess an alignment by comparing the paired coordinates directly, demonstrating that sequence alignment can be performed through (independent) Map functions.

["ac", "ag"]

which corresponds to the two axis of the original Chaos Game Representation (CGR) square [15]. In this example, the cube mapping [26] by the encoding operation [27] identified of the corners of a 2D plane as being 'a' → [0, 0], 'c' → [0, 1], 'g' → [1, 0], 't' → [1, 1]. The result of encoding the illustrative sequence above is displayed in Table 1 (note detail of where in the usm structure can the results be found) and Figure 1. The circular application (Figure 2) of Equtions1-2 can be verified by noting that the forward coordinates in the first row are at half the distance between the coordinates in the last row and the identity corners. The reverse happens for the backward coordinates: those in the last row are at half the distance between the coordinates in the first row and the identity corners.

**Table 1:** Numerical computation of USM encoding by generating forward (Equation 1) and backward (Equation 2) CGR successions.

***[ubase.bin,ubase.cgrForward,ubase.cgrBackward]***
["a", [0, 0], [0.0906, 0.2994], [0.2135, 0.3036]]
["c", [0, 1], [0.0453, 0.6497], [0.4271, 0.6072]]
["g", [1, 0], [0.5226, 0.3248], [0.8543, 0.2145]]
["g", [1, 0], [0.7613, 0.1624], [0.7086, 0.4290]]
["c", [0, 1], [0.3806, 0.5812], [0.4173, 0.8580]]
["t", [1, 1], [0.6903, 0.7906], [0.8347, 0.7161]]
["g", [1, 0], [0.8451, 0.3953], [0.6695, 0.4322]]
**["c", [0, 1], [0.4225, 0.6976], [0.3390, 0.8645]]**
["t", [1, 1], [0.7112, 0.8488], [0.6781, 0.7291]]
["a", [0, 0], [0.3556, 0.4244], [0.3563, 0.4582]]
["t", [1, 1], [0.6778, 0.7122], [0.7127, 0.9164]]
["c", [0, 1], [0.3389, 0.8561], [0.4254, 0.8328]]
["t", [1, 1], [0.6694, 0.9280], [0.8508, 0.6656]]
["g", [1, 0], [0.8347, 0.4640], [0.7016, 0.3312]]
["c", [0, 1], [0.4173, 0.7320], [0.4033, 0.6624]]
["g", [1, 0], [0.7086, 0.3660], [0.8067, 0.3248]]
["t", [1, 1], [0.8543, 0.6830], [0.6134, 0.6497]]
["a", [0, 0], [0.4271, 0.3415], [0.2269, 0.2994]]
["c", [0, 1], [0.2135, 0.6707], [0.4539, 0.5988]]
["g", [1, 0], [0.6067, 0.3353], [0.9079, 0.1976]]
["g", [1, 0], [0.8033, 0.1676], [0.8158, 0.3953]]
["t", [1, 1], [0.9016, 0.5838], [0.6316, 0.7907]]
["c", [0, 1], [0.4508, 0.7919], [0.2633, 0.5814]]
["g", [1, 0], [0.7254, 0.3959], [0.5266, 0.1629]]
["a", [0, 0], [0.3627, 0.1979], [0.0533, 0.3259]]
["c", [0, 1], [0.1813, 0.5989], [0.1067, 0.6518]]

See figure 1 for a graphical representation of the same succession. Note location of results in usm structure in the table's head.

### 2. Decoding

As described elsewhere [17,19,20], and can be verified in the accompanying tool, the value of each of the individual coordinates can be decoded bijectively by using equation 3 to map them back to a sequence. For example, starting with the 7th forward coordinates, highlighted in Table 1 a cytosine, "c", the binary source binary sequence for each of the CGR dimensions can be extracted. To make this illustration more compelling, lets starts with an instance of the usm object that is devoid of any sequence information (null) beyond the alphabet ('acgt'):

u = new usm(null,'acgt')

u.decodeBin(0.4225834224013004)

[0, 1, 1, 0, 1, 1, 0, 0, 0, 0, 1, 0, 1, 1, 1, 0, 0, 1, 1, 0, 1, 1, 0, 1, 0, 1, 0, 1, 1, 0, 1, 1, 0, 0, 0, 0, 1, 0, 1, 1, 1, 0, 0, 1, 1, 0, 1, 1, 0, 1, 0, 1]

u.decodeBin(0.6976523056276487)

[1, 0, 1, 1, 0, 0, 1, 0, 1, 0, 0, 1, 1, 0, 0, 1, 0, 1, 0, 1, 0, 1, 1, 1, 0, 1, 1, 0, 1, 1, 0, 0, 1, 0, 1, 0, 0, 1, 1, 0, 0, 1, 0, 1, 0, 1, 0, 1, 1, 1]

The decoding process maps the numeric coordinates back to the symbolic sequence by identifying the identity cube edges. Therefore, by applying it to the full coordinate vector one will retrieve the original sequence, both preceding (forward map) and succeeding (backward map) the coordinate position for that "c":

u.decode([0.4225834224013004, 0.6976523056276487]).reverse()

"tctgcgtacggtcgac.acggctgctatctgcgtacggtcgac.**acggctg[c]**"

u.decode([0.3390888473255761, 0.8645502159677478])

"**[c]tatctgcgtacggtcgac**.acggctgctatctgcgtacggtcgac.acggct"

This example, because it was performed on a sequence that is shorter than resolution of the CGR coordinates, can also be analyzed to illustrate the circular seeding procedure described in [19]. To make the decoded sequence clearer, a period (".") was inserted to indicate the origin position, where the two ends of the sequence were stitched together in the seeding process. It can then be confirmed that indeed the dynamic circular seeding procedure will generate cyclic images of the original sequence. This decoding operation also illustrates the extensive context information stored in a single pair of coordinates: the picture represented in Figure 2 could be built directly from the USM coordinates of each and any of this sequence's units.

### 3. Distance

A number of distance metrics have been identified by us and by other authors [17,21,22] that calculate the length of the similar segment shared by two units in two distinct sequences. As in those reports, the word "distance" will be used as short form for "dissimilarity distance metric", which is really a measure of similarity - the higher the value of the "distance" the higher the similarity. The defining feature of CGR derived distance metrics, and the reason for betting on them as replacements for the less scalable dynamic programming alignment procedures, is that they rely solely on the coordinates of the two sequence units being compared. Here we will use the formulation in Equation 3 as can be verified by inspecting the coding of method L in [25]. For example, in the comparison of two sequences from a binary alphabet (corners 0 and 1 in the real axis) with coordinates 0.01 and 0.001:

u.L(0.01, 0.001)

**Figure 3:**
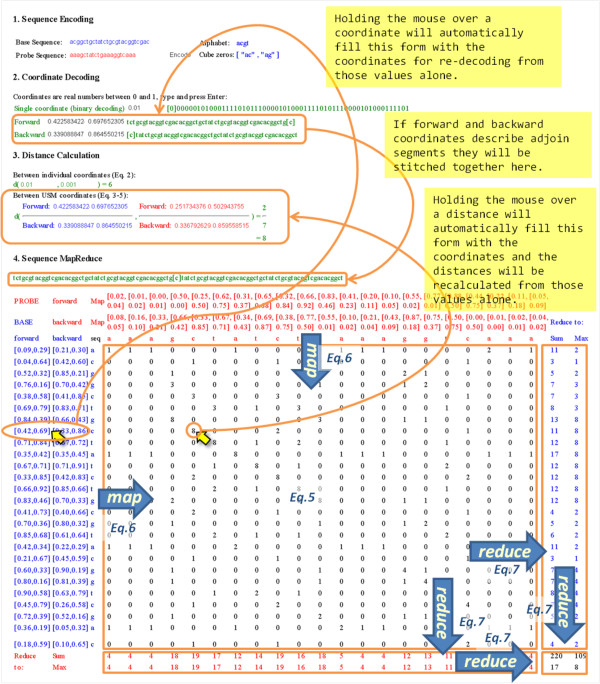
**Annotated snapshot of using the companion webApp at usm.github.com to run the examples used to illustrate Equations 5, 6 and 7**. The code hosting project site cgr.googlecode.com includes a tutorial and a video also describing the command line use of the libraries implementing the map-reduce decomposition of sequence analysis.

6

one finds out that they are at the end of a similar sub-sequence of length 6, their common prefix. The accuracy of this result, obtained without inspecting the coordinates of the preceding units, can be verified by independently decoding them into symbolic sequences: 000000101000111... and 000000000100000110..., confirming that the length of the shared sequence of 6 zeros was correctly imputed. This illustrative exercise can be done using u.decodeBin [28] as described in the Decoding section or, more conveniently, using the single coordinate decoding in the accompanying web tool (Figure 3).

### CGR distance - beginning of MapReduce decomposition

Because similar sequence can be determined directly for the coordinates of individual units, an expanded implementation of *L *(Equation 3) can now be produced (Equation 4) that takes advantage of the MapReduce parallelization pattern. The distance *d_cgr _*between two coordinates *c^a ^*and *c^b ^*is:

(4) $$\begin{array}{c} {\text{d}}_{\text{CGR}}\text{(}{\text{c}}^{\text{a}}\text{,}{\text{c}}^{\text{b}}{\text{) = \{c}}_{1}^{\text{a}}{\text{,c}}_{1}^{\text{b}}\text{],}...{\text{,[c}}_{\text{n}}^{\text{a}}{\text{,c}}_{\text{n}}^{\text{b}}]].\text{map }\left(\left(\left[{\text{c}}_{\text{i}}^{\text{a}},{\text{c}}_{\text{i}}^{\text{b}}\right]\right)\to \text{L}\left(\left[{\text{c}}_{\text{i}}^{\text{a}},{\text{c}}_{\text{i}}^{\text{b}}\right]\right)\right).\text{reduce}\left(\left(\text{a,b}\right)\to \text{min}\left(\left[\text{a,b}\right]\right)\right)\\ with\;i=1,...,n,\;where\;n\;is\;the\;number\;of\;dimensions\;of\;the\;CGR\;cube. \end{array}$$

As described in this equation, and can also be verified by inspecting [29], the procedure consists of calculating the *L *distance between each pair of coordinates (a **map **operation) and then take the minimum value of the resulting array (a **reduce **operation). It is worth comparing this equation with the code referenced by [29] to verify how closely the implementation is to the formulation:

*this.distCGR = function (a, b){*

*var dist = this.L;*

*return this **. transpose([a, b]).map(function(x){return dist(x[0], x[1])}).min();***

*}*

### USM distance

USM (bidirectional) coordinates, *[c^forward^, c^backward^]*, for a given sequence position consist of a pair of unidirectional CGR coordinates, determined forwardly (Equation 1) and backwardly (Equation 2). Therefore the indexes *forward *and *backward *indicate *n *numerical values each, as many as dimensions of the USM cube. Elaborating on the probabilistic metric proposed in [17], the CGR forward and backward distances are combined here to compute the exact similar length, in either direction, shared by two homologous units. This exact sequence dissimilarity distance metric is a novel result, and represents the length of the shared similar segment:

(5) $$\begin{array}{c} {\text{d}}_{\text{USM}}\left({\text{C}}_{\text{a}},{\text{C}}_{\text{b}}\right)={\text{d}}_{\text{CCR}}^{\text{forward}}\left({\text{C}}_{\text{a}}^{\text{forward}},{\text{C}}_{\text{b}}^{\text{forward}}\right)+{\text{d}}_{\text{CCR}}^{\text{backward}}\left({\text{C}}_{\text{a}}^{\text{backward}},{\text{C}}_{\text{b}}^{\text{backward}}\right)-1\\ for\;unidentical\;units\;a,b,{(\text{d}}_{\text{USM}}\left({\text{C}}_{\text{a}},{\text{C}}_{\text{b}}\right)=-1)\to \left({\text{d}}_{\text{USM}}\left({\text{C}}_{\text{a}},{\text{C}}_{\text{b}}\right)=0\right) \end{array}$$

Equation 5 is encoded verbatum in [30]. To clarify the calculation of *d_USM _*, a second sequence will now be encoded to be compared (to probe) with the base sequence used above to illustrate encoding. Close inspection of the probe sequence will reveal two segments that are also found in the base sequence, one with length 8 and the other with length 4. This example will be used to illustrate the shared similar segment determination using Equation 5:

*uprobe = new usm('aaagctatctgaaaggtcaa',ubase.abc)*

*> usm*

As *ubase*, *uprobe *is an instance of the usm object but its creation took an additional input argument, the alphabet identified for ubase. Although this was not necessary in the particular case of the probe sequence because the probe alphabet used the same four nucleotide alphabet, by providing the base alphabet as a second input argument the new encoding is guaranteed to be framed by the exact same hyper-dimensional binary cube. As with Table 1 for the base sequence, the encoded coordinates for the probe sequence are now provided in Table 2.

**Table 2:** Encoding of a second sequence to compare (probe) with the base sequence encoded in Table 1.

***[uprobe.bin,uprobe.cgrForward,uprobe.cgrBackward]***
["a", [0, 0], [0.0277, 0.0471], [0.0835, 0.0537]]
["a", [0, 0], [0.0138, 0.0235], [0.1670, 0.1074]]
["a", [0, 0], [0.0069, 0.0117], [0.3341, 0.2148]]
["g", [1, 0], [0.5034, 0.0058], [0.6683, 0.4297]]
***["c", [0, 1], [0.2517, 0.5029], [0.3367, 0.8595]]***
["t", [1, 1], [0.6258, 0.7514], [0.6735, 0.7191]]
["a", [0, 0], [0.3129, 0.3757], [0.3471, 0.4382]]
["t", [1, 1], [0.6564, 0.6878], [0.6943, 0.8764]]
["c", [0, 1], [0.3282, 0.8439], [0.3886, 0.7529]]
["t", [1, 1], [0.6641, 0.9219], [0.7773, 0.5058]]
["g", [1, 0], [0.8320, 0.4609], [0.5547, 0.0117]]
["a", [0, 0], [0.4160, 0.2304], [0.1094, 0.0234]]
["a", [0, 0], [0.2080, 0.1152], [0.2189, 0.0469]]
["a", [0, 0], [0.1040, 0.0576], [0.4378, 0.0939]]
["g", [1, 0], [0.5520, 0.0288], [0.8756, 0.1879]]
["g", [1, 0], [0.7760, 0.0144], [0.7513, 0.3758]]
["t", [1, 1], [0.8880, 0.5072], [0.5026, 0.7516]]
["c", [0, 1], [0.4440, 0.7536], [0.0052, 0.5033]]
["a", [0, 0], [0.2220, 0.3768], [0.0104, 0.0067]]
["a", [0, 0], [0.1110, 0.1884], [0.0208, 0.0134]]
["a", [0, 0], [0.0555, 0.0942], [0.0417, 0.0268]]

If the two "c" units in the base and probe sequences, positions 14th and 5th, highlighted, respectively, in Table 1 and Table 2 were to be compared by this method, only their USM coordinates would be needed to determine their distance, d*_USM_*., defined as the length of the shared similar segment. The step by step calculation of the coordinates for the two positions is reviewed in Table 3.

**Table 3:** Detailed calculation of length of similar segment, *d_USM_*, from USM coordinates of individual homologous units.

Encoding	
*ubase = new usm('acggctgctatctgcgtacggtcgac')*	
*uprobe = new usm('aaagctatctgaaaggtcaaa',ubase.abc)*	
*u = new usm(null, 'acgt')*	
Reviewing coordinates of positions highlighted in Table 1 and 2	
*ubase.cgrForward*[7]	*uprobe.cgrForward*[4]
*[0.4225834224013004, 0.6976523056276487]*	*[0.2517343767806896, 0.502943755599859]*
*ubase.cgrBackward*[7]	*uprobe.cgrBackward*[4]
*[0.3390888473255761, 0.8645502159677478]*	*[0.33679262961989864, 0.8595585153381897]*

**Calculating one step at a time**, $d_{CGR}^{forward}$**and **$d_{CGR}^{backward}$	
u.distCGR([0.4225834224013004, 0.6976523056276487],[0.2517343767806896, 0.502943755599859]) 2	
u.distCGR([0.3390888473255761, 0.8645502159677478],[0.33679262961989864, 0.8595585153381897]) 7	
**Applying Equation 5 directly to find length of similar segment = 2+7-1 = 8**	
u.dist(ubase.usm[7],uprobe.usm[4])8	

In this illustrative example, the coordinates for base and probe sequences for nucleotide "c" in position 8 and 5 respectively: acggct**g[c]tatctg**cgtacggtcgac, and aaa**g[c]tatctg**aaaggtcaaa will be compared using Equation 5. Note array indexes in JavaScript start with 0 (zero), so this corresponds to comparing coordinate indexes 7 and 4. This distance result is also highlighted in Figure 3.

### 4. USM MapReduced to compare full sequences

The MapReduce decomposition described in Equation 5 can be encapsulated in one more MapReduce parallelization operation to tabulate the comparison between full sequences (Eq. 6). The map component is straightforward application of Equation 5, and the reduce operation define the statistics that characterize the probing of the base sequence:

(6) $$\begin{array}{c} dMap\left(seq^{base},seq^{probe}\right)=USM^{base}.\;map\left(\left(x\right)\to \left(USM^{probe}.\;map\left(\left(y\right)\to \left(d_{USM}\left(x,y\right)\right)\right)\right)\right)\\ where\;\;USM=\left[\left[C^{forward}\right],\left[C^{backward}\right]\right] \end{array}$$

(7) $$\begin{array}{c} d\left(seq^{base},seq^{probe}\right)\\ \;=dMap\left(seq^{base},seq^{probe}\right).\;reduce\left(\left(x,y\right)\to S\left(x,y\right)\right).\;reduce\left(\left(x,y\right),\to ,S,\left(x,y\right)\right)\\ where\;S\;is\;some\;statistic\;on\;a\;pair\;of\;values,such\;as\;sum\;or\;maximum. \end{array}$$

In the accompanying web-tool, this is illustrated with both order statistics (length of maximum common segment) and parametric statistics (sum of lengths). A snapshot of the use of this tool to run the examples used as illustrations in this section is depicted and annotated in Figure 3. The coding of Equation 6 as two mapping operations populating a 2D array [31] is almost exactly as in the formulation. The only additional consideration is that a different encoded base sequence could be provided as a second input argument. Using the example in Table 3 these two expression would produce the same result: ubase.distMap(uprobe), or u.distMap(uprobe, ubase).

ubase.distMap(uprobe)

[

[1, 1, 1, 0, 0, 0, 1, 0, 0, 0, 0, 1, 1, 1, 0, 0, 0, 0, 2, 1, 1],

[0, 0, 0, 0, 1, 0, 0, 0, 1, 0, 0, 0, 0, 0, 0, 0, 0, 1, 0, 0, 0],

[0, 0, 0, 1, 0, 0, 0, 0, 0, 0, 1, 0, 0, 0, 2, 1, 0, 0, 0, 0, 0],

[0, 0, 0, **3**, 0, 0, 0, 0, 0, 0, 1, 0, 0, 0, 1, 2, 0, 0, 0, 0, 0],

[0, 0, 0, 0, **3**, 0, 0, 0, **3**, 0, 0, 0, 0, 0, 0, 0, 0, 1, 0, 0, 0],

[0, 0, 0, 0, 0, **3**, 0, 1, 0, 3, 0, 0, 0, 0, 0, 0, 1, 0, 0, 0, 0],

[0, 0, 0, **8**, 0, 0, 0, 0, 0, 0, **3**, 0, 0, 0, 1, 1, 0, 0, 0, 0, 0],

[0, 0, 0, 0, **8**, 0, 0, 0, 2, 0, 0, 0, 0, 0, 0, 0, 0, 1, 0, 0, 0],

[0, 0, 0, 0, 0, **8**, 0, 1, 0, 2, 0, 0, 0, 0, 0, 0, 1, 0, 0, 0, 0],

[1, 1, 1, 0, 0, 0, **8**, 0, 0, 0, 0, 1, 1, 1, 0, 0, 0, 0, 1, 1, 1],

[0, 0, 0, 0, 0, 1, 0, **8**, 0, 1, 0, 0, 0, 0, 0, 0, 2, 0, 0, 0, 0],

[0, 0, 0, 0, 2, 0, 0, 0, **8**, 0, 0, 0, 0, 0, 0, 0, 0, 2, 0, 0, 0],

[0, 0, 0, 0, 0, 2, 0, 1, 0, **8**, 0, 0, 0, 0, 0, 0, 1, 0, 0, 0, 0],

[0, 0, 0, 2, 0, 0, 0, 0, 0, 0, **8**, 0, 0, 0, 1, 1, 0, 0, 0, 0, 0],

[0, 0, 0, 0, 2, 0, 0, 0, 1, 0, 0, 0, 0, 0, 0, 0, 0, 1, 0, 0, 0],

[0, 0, 0, 1, 0, 0, 0, 0, 0, 0, 1, 0, 0, 0, 1, 2, 0, 0, 0, 0, 0],

[0, 0, 0, 0, 0, 2, 0, 1, 0, 1, 0, 0, 0, 0, 0, 0, 2, 0, 0, 0, 0],

[1, 1, 1, 0, 0, 0, 2, 0, 0, 0, 0, 1, 1, 1, 0, 0, 0, 0, 1, 1, 1],

[0, 0, 0, 0, 1, 0, 0, 0, 1, 0, 0, 0, 0, 0, 0, 0, 0, 1, 0, 0, 0],

[0, 0, 0, 1, 0, 0, 0, 0, 0, 0, 1, 0, 0, 0, **4**, 1, 0, 0, 0, 0, 0],

[0, 0, 0, 1, 0, 0, 0, 0, 0, 0, 1, 0, 0, 0, 1, **4**, 0, 0, 0, 0, 0],

[0, 0, 0, 0, 0, 1, 0, 2, 0, 1, 0, 0, 0, 0, 0, 0, **4**, 0, 0, 0, 0],

[0, 0, 0, 0, 1, 0, 0, 0, 2, 0, 0, 0, 0, 0, 0, 0, 0, **4**, 0, 0, 0],

[0, 0, 0, 1, 0, 0, 0, 0, 0, 0, 2, 0, 0, 0, 1, 1, 0, 0, 0, 0, 0],

[1, 1, 1, 0, 0, 0, 1, 0, 0, 0, 0, 2, 1, 1, 0, 0, 0, 0, 1, 1, 1],

[0, 0, 0, 0, 1, 0, 0, 0, 1, 0, 0, 0, 0, 0, 0, 0, 0, 2, 0, 0, 0]

]

Using Equation 7 as a template, the maximum shared segment between the two sequences would then be done with two reduce operations:

S = function(x, y){return [x, y].max()};

ubase.distMap(uprobe).reduce(S).reduce(S)

8

It is also interesting to note that the 2D distance map would not have to be fully resolved to find out what is the maximum similar length. Since that value, *d_USM _*, can be determined from any pair of homologous units, a result of *L *only requires that every *L^th ^*be analyzed.

### 5. Sequence alignment to full genomes

Although the results described in the previous 4 sections and the accompanying webApp describe the decomposition of the fractal encoding and decoding USM procedure, the ultimate test is, as the title hints, the ability to align biological sequences. For this test to be conclusive, it should also establish that there are no fundamental issues that would prevent scaling it to the processing of long sequences. As in the other 4 sections, the results described in this one relies exclusively on the browser's computational environment. As before, a webcast of the procedure was also included (see Video #2 link in the webApp).

### Loading and processing full genomes

Two genomes will be used to demonstrate the procedure, the small genome of *Streptococus sp*. Phage 2972 (NC007019, gi 66391759), which has close to 34 Kbp, and the first full genome of a strain of its notorious host, *Streptococcus pneumomiae *R6 (NC003098, gi 15902044), with over 2 million base pairs. As in section 1, loading and processing the sequence is handled automatically by the instantiation of the USM object. The syntax is the same except that we will use the URL of the fastA file with the full genome rather than the raw sequence:

uPhage = new usm('http://ftp:/ / ftp.ncbi.nlm.nih.gov/ genomes/ Viruses/ Streptococcus_phage_2972_uid15254/ NC_007019.fna')

which takes approximately 4 seconds to load and process by the USM procedure, with the browser using approximately 40 Mb or RAM;

> uBac = newusm('ftp://ftp.ncbi.nlm.nih.gov/genomes/Bacteria/Streptococcus_pneumoniae_R6_uid57859/NC_003098.fna')

which takes approximately 15 seconds to load and process, with the browser using a little over 1/2 GB of RAM while going through the USM indexing procedure. These numbers were obtained using Google's Chrome web browser running on a modestly resourced MacBook Air laptop (1.8 GHz CPU 4 GB RAM). It is also noteworthy that no attempt was made to optimize memory usage by storing away USM indexing results as they are being produced. The screencast of these tests is provided as "Video#2" in the webApp. The exact times will depend on the machine and connection available but these are values that establish the USM procedure, and iterated maps in general, as not representing an un-scalable route to sequence analysis.

### Alignment

The *distMap *illustration in the previous section of how to obtain the full USM distance map (Equation 6) between two sequences suggests that new alignment algorithms can be devised to make full use of that property that each of these distances can be obtained independently of each other. For example, each of the distance diagonals in that illustration identifies a square where all distance values are smaller than the diagonal. Therefore, resolving a single distance value in the diagonal automatically removes the need to resolve the rest of the square. The USM library includes an alignment method, to illustrate this procedure. Applying it to the two short sequences mapped above will readily align them by the position of the longest similar segment:

new usm('acggctgctatctgcgtacggtcgac').align('aaagctatctgaaaggtcaaa')

largest identical segment has length 8 and aligns with position 6 in base

sequence and position 3 in probe sequence

However, since CGR procedures are limited by the numerical resolution of the processor, additional steps will be needed to deal with diagonals longer than what can be resolved from a single value. A simple solution is found by repeating the dissimilarity distance calculation at the edges of the resolvable region. In summary, determining the identity between long segments should only require the resolution of one out of each 64 × 64 = 4096 map distance values. Not only can sequence similarity be decomposed into independent (parallelized) distance calculations, but also only a small fraction of those calculations are actually needed to resolve the distance map. Let us start by extracting a longer sequence from the phage genome than could possibly be resolved by a single comparison between two USM coordinates. Note this segment is made of by flanking a 100 unit long segment with two distinct 20 unit long segments.

someSeq = uPhage.seq.slice(0, 20)+uPhage.seq.slice(30000, 30100)+uPhage.seq.slice (10000, 10020)

"GGTTCGAAAATTACATTAAGCCAATGACTGAAAACGACATTCGGAGGGTGTGGCGAGATAATCCAGATGCTAACATTGCACTTAGAACAGATACATTCTTTGTCATTGACGTGGACATGCCATACGTTGTTGAAGAAGCT"

Let us now align it back to the original sequence:

A = uPhage.align(someSeq)

largest identical segment has length 113 and aligns with position 30000 in

base sequence and position 20 in probe sequence

This determination is nearly instantaneous, and close inspection of the A structure will show that the alignment required a single step. Inspection of the align method in the source code of the library will reveal that the 100 long segment is resolved by extending the diagonal with distance calculation 64 positions apart in the diagonal. It is also interesting how this alignment procedure can be used to identify multiple matches. For example,

A = uBac.align('TCCACAGCATGCGTGACGATGACACG')

will produce three 10 unit long matches within the 2 Mbp pneumococcal genome, at positions 1811967 ("AGCATGCGTG"), 1895547 ("TCCACAGCAT") and 1992091 ("GACGATGACA").

## Discussion

In [17] we first noted that by adding the distances of forwardly and backwardly encoded coordinates we could estimate the length of the full similar segment. This had the interesting property that the length of the similar sequence could be approached by comparing the forward and backward coordinates of any two homologous units. This is the defining feature explored by the map-reduce decomposition described here. This composite of forward and backward CGR coordinate encoding for alphabets of any length was designated as Universal Sequence Maps (USM). A compact library [usm.m] was then developed in Mathworks m-code to support motif density kernels [19]. The library provided here (usm.js) advances that work by producing exact measure of similar length (Equations 3-5), and weaving its use in Equation 6 with MapReduce parallelization of sequence comparison.

**Figure 4:**
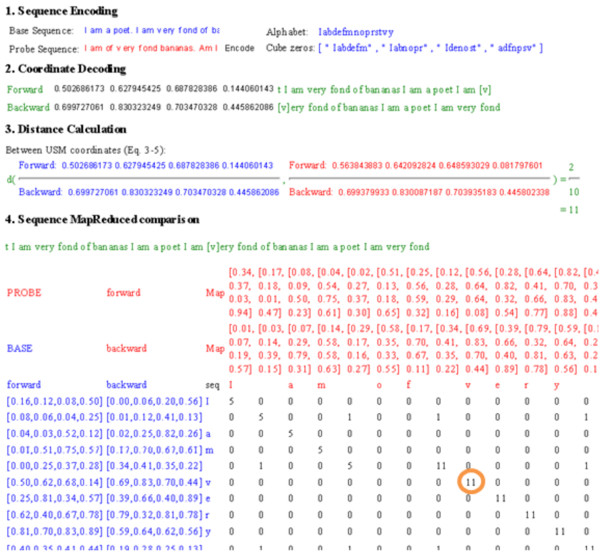
**Non-genomic sequence comparison example borrowed from [Almeida 2002] of encoding a non-genomic sequence**. See Figure 3 for notes on the layout of the web tool.

The two preceding reports discussed above, as well as a more recent exploit [20], sought to expand the CGR solution [15] to arbitrary alphabets. Although the examples of map-reduce decomposition in the Results section offer illustrations for genomic data, the formulations, accompanying libraries and webApp are just as applicable to other types of symbolic sequence. For example, using the illustrative sequence comparison used in [17], "I am a poet. I am very fond of bananas" and "I am of very fond bananas. Am I a poet", two stanzas borrowed by a poem by Wendy Cope, the same decomposition will encode those sequences in a 4-dimension CGR/USM space (Figure 4). As that figure demonstrates, the decoding and the computation of distance between sequences use the exact same USM procedure, and the exact same libraries reported here. In summary, the procedures described here are applicable to sequences of any alphabet.

The accompanying USM library was developed primarily to demonstrate the decomposition of sequence analysis allowed by this representation. However this is not free of computational costs. In order to realize the analytical advantages of the USM procedure, the sequences have to be pre-processed/indexed by the CGR iterated function. Nevertheless, as detailed in section 5 of Results, processing small genomes is actually achieved in only a few seconds in the web browser of a modestly resourced laptop. That section, under "alignment", also highlights another surprising feature of the iterated map representation. Although they are normally described as "alignment-free", the decomposition described here actually offers a very efficient route for aligning sequences. This was demonstrated by aligning sequences of various lengths by the positions of their longest identical segments. Further elaborating on this result by developing iterated map equivalents of well-established alignment algorithms for local/global, and with/without recursion features, is beyond the scope of this report, but it now becomes a distinct possibility.

The use of JavaScript to develop an implementation of the alignment-free [5] map-reduce decomposition of sequence analysis could be justified solely on grounds of convenience. Since web browsers are equipped with JavaScript interpreters, that is their native language, and as the accompanying webApp demonstrates, the js library developed can be conveniently distributed without requiring "download", "installation" or "updates". In modern browsers an argument can also be made for the efficient performance of this environment as a computational engine. For example, modern js compilers will automatically recognize opportunities to use Graphic Processing Units (GPU). A third argument can be added, regarding the amenability of js to functional programming styles. As described by one of its principal curators, Douglas Crockford, "JavaScript is LISP with C's clothing" [32]. Its functional nature invites the development of interpreters for higher-level domain specific languages, including some that mimic mathematical notation, such as coffeesript http://coffeescript.org. The last argument may be the most consequent, even if it does not presently lend itself to practical verification. It is not a stretch of imagination to expect the web's configuration as a high performance computing (HPC) environment that relies on code migration: unlike data, JavaScript code migration is not affected by same-domain origination restrictions. At some point in the future, the web may offer efficient distributed map-reduce constructs that span multiple browsers running in multiple machines. Until then, we are left with more conventional distributed MapReduce environments, such as Hadoop and MongoDB, where to attempt scalable deployment of the sequence analysis procedures reported here.

## Conclusions

The Universal Sequence Map (USM) procedure expands the Chaos Game Representation (CGR) approach to "alignment-free" analysis of sequences of any alphabet. Not only is the sequence comparison procedure described here performed without recourse to dynamic programming alignment, but multiple layers of nested map-reduce distribution provide maximally parallelized workflows to find the length of the similar segment shared by any two sequence units. If this basic alignment operation can be streamlined by the USM procedure into the scalable and distributed processing form described here, the expectation is that other sequence analysis operations can be similarly decomposed, including more advanced types of alignment proceedures. This may be particularly significant given the large amount of sequence information now being generated by NextGen methodologies. The proposed MapReduce decomposition was implemented in "the language of the web", JavaScript (ecmascript), both out of convenience and in arguable anticipation of the native use of web-browsers for distributed computing.

## Competing interests

The authors declare that they have no competing interests.

## Authors' contributions

JSA identified and implemented the USM decomposition procedure; AG assisted with the use of the Map Reduce programming pattern, with oversight by WM in framing it in the wider context of computational architectures; SV was integral part of the re-design of the USM procedure, with a key contribution in its mathematical representation. JSA wrote the report and all authors participated in its revision.
